# Coverage and Financial Risk Protection for Institutional Delivery: How Universal Is Provision of Maternal Health Care in India?

**DOI:** 10.1371/journal.pone.0137315

**Published:** 2015-09-08

**Authors:** Shankar Prinja, Pankaj Bahuguna, Rakesh Gupta, Atul Sharma, Saroj Kumar Rana, Rajesh Kumar

**Affiliations:** 1 School of Public Health, Post Graduate Institute of Medical Education and Research, Chandigarh, India; 2 National Rural Health Mission, Department of Health and Family Welfare, Haryana, India; Groningen Research Institute of Pharmacy, NETHERLANDS

## Abstract

**Background:**

India aims to achieve universal access to institutional delivery. We undertook this study to estimate the universality of institutional delivery care for pregnant women in Haryana state in India. To assess the coverage of institutional delivery, we analyze service coverage (coverage of public sector institutional delivery), population coverage (coverage among different districts and wealth quintiles of the population) and financial risk protection (catastrophic health expenditure and impoverishment as a result of out-of-pocket expenditure for delivery).

**Methods:**

We analyzed cross-sectional data collected from a randomly selected sample of 12,191 women who had delivered a child in the last one year from the date of data collection in Haryana state. Five indicators were calculated to evaluate coverage and financial risk protection for institutional delivery—proportion of public sector deliveries, out-of-pocket expenditure, percentage of women who incurred no expenses, prevalence of catastrophic expenditure for institutional delivery and incidence of impoverishment due to out-of-pocket expenditure for delivery. These indicators were calculated for the public and private sectors for 5 wealth quintiles and 21 districts of the state.

**Results:**

The coverage of institutional delivery in Haryana state was 82%, of which 65% took place in public sector facilities. Approximately 63% of the women reported no expenditure on delivery in the public sector. The mean out-of-pocket expenditures for delivery in the public and private sectors in Haryana were INR 771 (USD 14.2) and INR 12,479 (USD 229), respectively, which were catastrophic for 1.6% and 22% of households, respectively.

**Conclusion:**

Our findings suggest that there is considerably high coverage of institutional delivery care in Haryana state, with significant financial risk protection in the public sector. However, coverage and financial risk protection for institutional delivery vary substantially across districts and among different socio-economic groups and must be strengthened. The success of the public sector in providing high coverage and financial risk protection in maternal health provides encouragement for the role that the public sector can play in universalizing health care.

## Introduction

India has witnessed a significant reduction in its maternal mortality ratio (MMR) from 437 per 100,000 live births in 1990 to 140 per 100,000 live births in 2015 [1]. There exist inter-regional variations in MMR reduction, with wealthier states likely to achieve the Millennium Development Goals (MDG) with only a short delay of 2–3 years [2, 3]. Achieving universal access to institutional delivery has been recognized as a major strategy for improving maternal survival. A delivery is said to be institutional if a woman delivers at a public, private or charitable trust/NGO health facility [4].

Maternal complications can be fatal in the absence of proper medical intervention, especially at the time of delivery and within the first 48 hours of the postpartum period [5, 6]. Despite improved coverage, i.e. utilization of services for institutional delivery during the last decade, social, physical, cultural and financial barriers to accessing health care exist. In our paper we refer to coverage as utilization of services, rather than any risk cover by a health insurance scheme. Almost one-fourth of women still deliver at home in India. The most common reasons cited for home deliveries are traditional practice and financial constraints [7]. Similar to the overall healthcare services in India, out-of-pocket payment remains the predominant source of financing for delivery services [8, 9]. An analysis of the District Level Household Survey (DLHS) reported that 48% of deliveries were unsafe in the absence of any medical supervision [10]. Those delivering in private facilities, undergoing Caesarean sections, and having higher educational and socio-economic status were more likely to incur high OOP expenses [10, 11]. Moreover, OOP expenditure on deliveries was catastrophic in 16% of the households surveyed.

To address the multiple barriers hindering access to health care services, the Government of India (GoI) introduced a series of programs under its flagship program, National Rural Health Mission (NRHM), now called as National Health Mission (NHM). These services include creation of village-level health workers called Accredited Social Health Activists (ASHAs), introduction of publicly financed referral transport, strengthening of service provision at Primary Health Centers (PHCs) around the clock, introduction of conditional cash transfers for institutional delivery—*Janani Surkaha Yojana* (JSY)—and implementation of a program for free cashless delivery in public institutions—*Janani Shishu Suraksha Karaykaram* (JSSK). The cadre of ASHA workers was created to generate demand at the community level for MCH services, including institutional delivery. While referral transport and strengthening of PHCs were intended to reduce physical barriers to access, the final two initiatives (i.e., JSY and JSSK) were implemented to reduce the financial barriers. Under the JSY scheme, cash incentives are given to women for delivering their babies in public sector health facilities or accredited private institutions in some states [12]. Under the JSSK scheme, all pregnant women delivering in public or accredited private health facilities are entitled to free delivery procedures (normal or Caesarean), medications, diagnostics, meals, provision of blood and transport services without any user charges [13]. These programs have been initiated under an overall policy directed toward Universal Health Care (UHC) in India. Achieving UHC has been stated as a goal by various policy documents sharing the vision for health in India [14, 15].

These programs for universalizing institutional delivery care have been evaluated to assess their impacts on the attainment of the desired targets [12, 16–20]. The JSY program has been reported to increase institutional delivery and reduce maternal deaths [12]. Another study reported a rise of more than 2.5 times in the institutional deliveries in district Faridabad in Haryana state during the post-JSSK period. A recent study that evaluated the implementation of the JSSK program among urban slum dwellers in Chandigarh City found a significant reduction in OOP expenditures (33%) but an insignificant reduction in catastrophic delivery expenditures in the post JSSK period [18, 19].

The existing literature assessing the impacts of different programs on institutional delivery and related OOP expenditures also has limitations. Most of the studies focused on individual interventions and did not assess the impact of the entire package. Moreover, the majority of the studies used the old databases, which cover a period up to 2008, whereas many changes have occurred during the last 5 years. Some of the studies that drew on recent data had limited sample sizes or were conducted in focal geographical areas among specific population subgroups and hence had limited generalizability. Against this background of gaps in evidence and the recent strides toward UHC, we undertook this study to estimate the universality of institutional delivery care for pregnant women in India’s Haryana state. To do so, we assessed coverage and patterns of institutional delivery care and financial risk protection against OOP expenditure on delivery. Second, we examined geographic- and wealth-based inequities in utilization of and OOP expenditures for institutional delivery care. Finally, to assess whether the benefits of maternal health interventions (i.e., making delivery in public facilities free of cost) are reaching the most disadvantaged, we assessed the determinants of “no expenditure on delivery” to provide insights for future policy measures.

## Methodology

### Study Setting

Haryana is one of the northern states in India. It falls in the top quartile in terms of per capita gross domestic product (GDP) in India. The overall population of the state is almost 25 million [21]. The Human Development Index (HDI) value of the state is 0.545 [22]. However, the state lags behind others in many health and healthcare service indicators. In terms of the infant mortality rate (42 per 1000 live births), Haryana ranks 27 among 35 states and Union Territories in India, which is surprisingly low considering the human and economic development in the state.

The state has 53 secondary and tertiary care hospitals, 95 Community Health Centers (CHCs), 440 PHCs and 2,630 Subcenters (SCs) for provision of health services [21]. Public health expenditure in the state was INR 483 (USD 8.1) per capita in 2012–13, of which state and center spending was INR 403 (USD 6.7) and INR 80 (USD 1.3) per capita, respectively [23, 24].

### Data Collection

This analysis is based on data collected as part of a large household survey undertaken in the state of Haryana to measure the extent of UHC. As part of this survey, 30 graduate-level field investigators collected household-level information from a randomly selected sample of Subcenters in all 21 districts of the state. A multistage stratified random sampling design was used in selection of primary sampling units (PSUs, i.e., Subcenters), villages, and households. In each PSU, 6 categories of individuals were interviewed—women who had delivered a baby within the last year, women with a child between 12–23 months of age, women with a child less than 5 years age, eligible couples (married couples with a woman between the ages of 15 and 45), those who had experienced an illness episode within the last 15 days, and those with a history of hospitalization within the last 365 days. These individuals were interviewed to collect information on utilization of services for maternal health, child health, family planning and curative care, respectively. Finally, we also collected data on out-of-pocket (OOP) expenses incurred at the point of service utilization.

**Fig 1:**
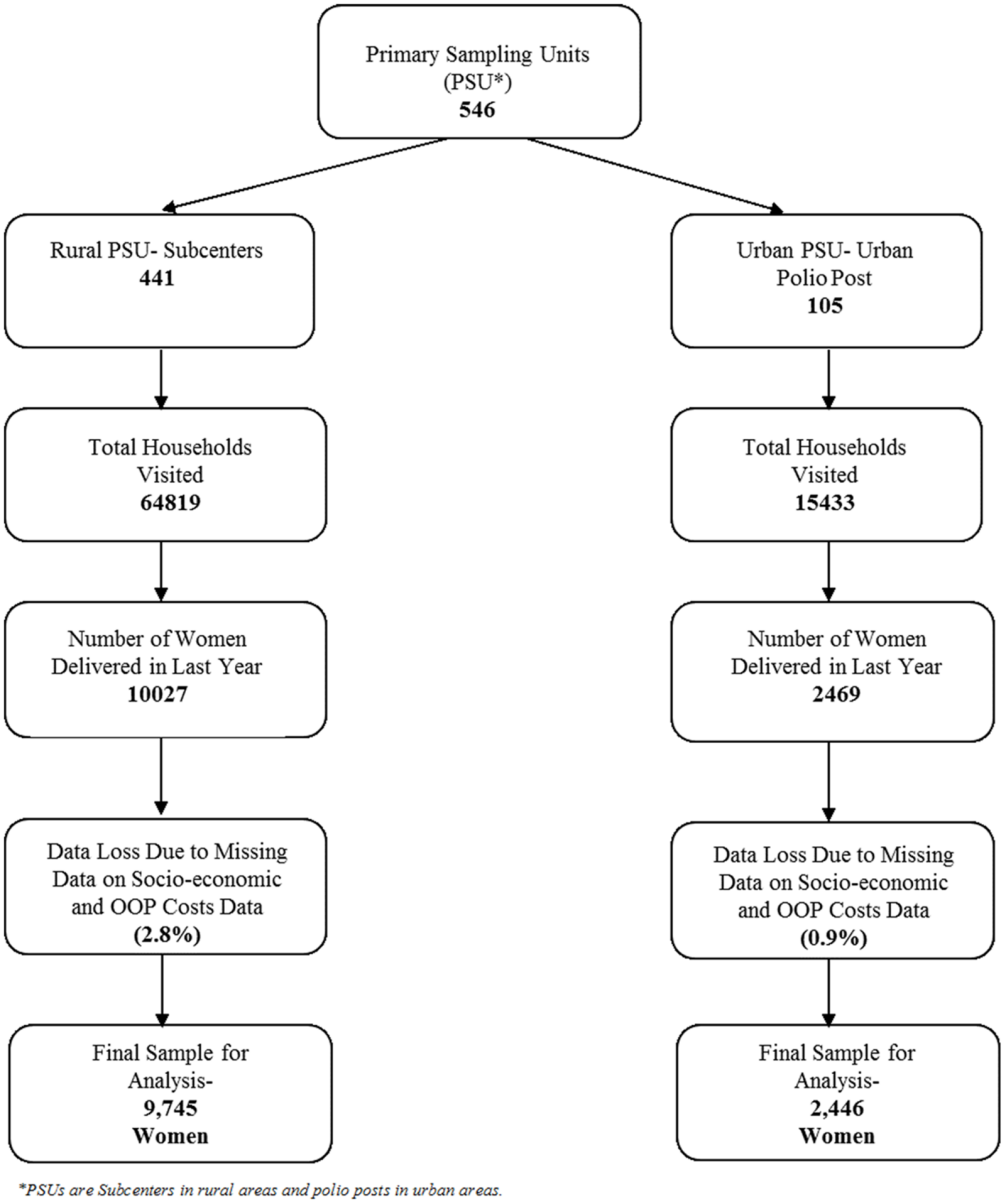
Flow Diagram Showing the Description of Data Collection.

In this paper, we present the analysis, which is based on data collected on utilization of institutional care at delivery that was administered to women who had delivered a baby in the last year. The data were collected during the period from September 2012 to March 2014. Hence, the data represent the extent of utilization and OOP expenditures for deliveries that occurred in Haryana from September 2011 to March 2014. The overall sample comprised 12,427 women who had delivered in the last year. The women were interviewed using 2 schedules—one for capturing their socio-demographic characteristics and consumption expenditures and the other for service utilization and its associated OOP expenditures. Some women had data missing for one of these variables (Fig 1). The final data used for analysis thus comprised 12,191 women.

### Data Analysis

#### Outcome Indicators

We calculated five indicators—the proportion of deliveries conducted at home and at public and private sector facilities, proportion of women incurring no OOP expenses on deliveries at public sector facilities, mean OOP expenditure on delivery, prevalence of catastrophic delivery expenditure and impoverishment headcount as a result of OOP expenditure on delivery. The overall percentage of institutional deliveries was assessed according to the type of institution (public or private); area; religion, caste, occupation and education of the head of the household; wealth quintile and BPL status of the household and type of delivery. Two thresholds have been suggested to estimate catastrophic health expenditures [25]. We categorized health care expenditure as catastrophic if it exceeded 40% of the household’s non-food expenditure. This method is viewed as better (than 10% of overall consumption expenditure) to capture the economic burden of health expenditure among poor individuals who spend a greater proportion of their total consumption expenditure on health [26–28]. The mean levels of OOP expenditures as percentages of total household and non-food expenditures were also calculated to examine the severity of the economic burden resulting from OOP expenditure on delivery. OOP expenditures in different years were adjusted for inflation and converted to 2013. We converted the costs in Indian National Rupees (INR) to United States Dollars (USD) using a conversion rate of one USD to INR 54.4 for the year 2013 [29, 30]. We also estimated the impoverishment induced as a result of OOP expenditure on delivery, which is defined as the percentage of households living below the poverty threshold. For this indicator, we calculated the poverty headcounts computing per capita household consumption expenditure before and after delivery. Two thresholds of poverty (i.e., international 1.25$ and 2$) were used. Households with per capita consumption expenditures below the given poverty thresholds were included in the poverty headcount. Finally, the difference between the pre- and post-delivery poverty headcounts provided the impoverishment as a result of OOP expenditures on delivery.

#### Equity Analysis

We divided the sample into five wealth quintiles based on households’ ownership of assets from a preset list of items [31–34]. The list included 27 items commonly used at the household level, such as computer, television, motorbike, car, mobile phone, refrigerator, chair, and table. The principal component analysis (PCA) method was used to assign a score to each household based on its ownership of assets. We ascertained the degree of inequality in service utilization and out-of-pocket payments.

Concentration Indices (CI) with 95% confidence limits were calculated for the indicators public sector institutional delivery, women with no expenditure on delivery and catastrophic delivery expenditure. The value of CI ranges from -1 to 1, where 0 implies equal utilization of health services across the population groups. A value between 0 and 1 implies higher service utilization among the wealthy population, whereas a value between -1 and 0 implies higher service utilization by the economically poor households.

#### Multivariate Analysis

We performed a multivariate regression using binary logit to assess the determinants of “no expenditure on delivery”. For the purpose of regression, the outcome variable (i.e., women or households that had incurred no expense in delivery) was coded as ‘1’ and the others as zero. The age of the mother; religion, caste, occupation and educational status of the head of household; socio-economic status of the family; and place and type of the delivery were used as independent variables in regression. Correlations between independent variables were assessed before introducing them in regression to rule out the presence of multi-collinearity. Standard regression technique, which allows introduction of all the independent variables in the model at the same time, was applied to identify associations of probable predictors with the outcome variable controlling the effect of the other variables. The odds ratio is reported as a measure of association along with the 95% confidence limits and p-value.

### Funding and Ethics

The study was funded by the National Rural Health Mission, Department of Health and Family Welfare, Government of Haryana. Ethical approval was granted by the Institute Ethics Committee of the Post Graduate Institute of Medical Education and Research, Chandigarh, India. We obtained written informed consent from respondents in all the households that were included in the survey. The administrative approval of the Health Department, Government of Haryana was also obtained.

## Results

### Sample Characteristics

We collected data on a randomly selected 12,469 women who had delivered a baby in the last year in Haryana. Due to missing information on socio-economic status and OOP expenditure, we analyzed data on 12,191 women (Fig 1). Sample characteristics are shown in Table 1. Of the 12,191 women interviewed in the reference period, the majority lived in rural areas (80%) and were Hindus (81%). Nearly 39% and 34% belonged to the Schedule Caste (SC) and Other Backward Class (OBC), respectively. The majority of the heads of households were literate (70%) and self-employed (66%). Almost 18% women belonged to families with below poverty line (BPL) status.

**Table 1:** Socio-Demographic Profile, Delivery Characteristics and Delivery Expenditures.

			No Expenditure on Delivery	Catastrophic Health Expenditure			Mean Out-of-Pocket Expenditure		
		Sample	Public	Public	Private	Home	Public	Private	Home
Characteristics		N (%)	%	%	%	%	Mean (SE*)	Mean (SE)	Mean (SE)
Overall		12191 (100)	63.7	1.6	22.0	0.7	771 (38)	12479 (270)	881 (68)
Area	Rural	9745 (79.9)	65.6	1.8	22.9	0.7	756 (40)	11901 (244)	897 (89)
	Urban	2446 (20.1)	52.1	0.7	20.4	0.8	867 (110)	14046 (751)	838 (81)
Religion	Hindu	9450 (80.9)	64.3	1.4	24.3	0.8	710 (38)	12525 (298)	694 (44)
	Muslim	1119 (9.6)	56.9	1.1	21.4	0.0	496 (68)	8491 (659)	794 (69)
	Christian	397 (3.4)	57.9	4.7	17.9	1.1	2138 (461)	11614 (1140)	2862 (1035)
	Sikh	703 (6.0)	58.6	2.6	10.3	2.4	2025 (444)	13280 (985)	2108 (662)
Caste	SC	4548 (39.2)	65.5	2.0	27.4	1.4	737 (54)	10339 (373)	674 (61)
	ST	43 (0.4)	48.1	5.0	25.0	0.0	865 (556)	12480 (3096)	185 (164)
	OBC	3988 (34.4)	60.2	1.4	20.7	0.2	731 (62)	12367 (569)	922 (120)
	General/Others	3024 (26.1)	62.9	1.0	19.6	0.6	948 (106)	13885 (402)	1296 (165)
Occupation	Self Employed	7680 (65.8)	62.4	1.7	23.8	0.7	732 (42)	11538 (350)	770 (77)
	Wage Employee and Others	646 (5.5)	80.4	2.1	31.6	0.0	639 (165)	12042 (1018)	2418 (449)
	Unemployed	1249 (10.7)	61.8	1.7	22.3	0.8	938 (152)	13153 (606)	1222 (304)
	Salaried Employee	2103 (18.0)	60.8	1.0	17.2	0.5	938 (117)	14177 (603)	699 (74)
Education	Illiterate	3464 (29.6)	63.3	1.4	25.3	1.1	751 (74)	10463 (381)	1024 (150)
	Literate	8242 (70.4)	63.3	1.7	20.9	0.5	793 (46)	12996 (330)	770 (49)
Wealth Quintile	Poorest	1786 (15.2)	67.9	2.0	41.2	1.2	633 (90)	10515 (648)	783 (77)
	Poor	2178 (18.6)	67.2	2.5	28.8	0.7	725 (81)	9846 (506)	688 (68)
	Moderate	2372 (20.2)	64.3	1.8	27.5	0.7	735 (72)	10637 (484)	756 (87)
	Rich	2597 (22.2)	63.1	1.4	22.4	0.4	773 (91)	11893 (423)	901 (113)
	Richest	2788 (23.8)	54.1	0.6	15.3	0.6	1024 (101)	14633 (557)	1439 (392)
BPL Status	BPL	2163 (17.7)	65.0	1.9	30.4	0.7	730 (74)	10653 (544)	671 (62)
	Non-BPL	10028 (82.3)	63.3	1.5	20.6	0.7	782 (43)	12715 (297)	928 (82)
Type of Delivery	Normal	10651 (87.5)	64.5	1.0	11.6	0.5	539 (25)	8479 (209)	787 (46)
	C-section	1516 (12.5)	55.6	6.6	51.7	33.3	3004 (307)	24958 (756)	24088 (9870)

*SE: Standard Error

Note: Stratum totals do not add up to overall total due to missing data.

### Service Utilization

The coverage of institutional delivery in Haryana state was 82%, of which 65% took place in public sector facilities (Table 2). There was no significant change in the quarter-wise (3-monthly blocks during the study period from September 2011 to March 2014) proportions of deliveries in the public and private sectors. The public sector was utilized for institutional delivery at a rate that was higher for the poorest (78%) than the richest (47%) wealth quintiles, which was significantly equitable (CI = -0.102). There were wide geographic variations, with the extent of public sector utilization for institutional delivery varying from 59% to 84% (Table 3).

**Table 2:** Equity in Care Utilization and Out-of-Pocket Expenditure for Delivery in Haryana State, India.

Characteristics	Wealth Quintile						
	Q1	Q2	Q3	Q4	Q5	Overall	Concentration Index
Public Sector Institutional Delivery (%)	78	77	70	63	47	65	-0.102 (-0.110, -0.093)
Women Incur No Expenditure on Delivery in Public Sector (%)	68	67	64	63	54	64	-0.040 (-0.05, -0.029)
***Mean Out-of-Pocket Expenditure (INR)***							
Public	633	725	735	773	1024	771	
Private	10515	9846	10637	11893	14633	12479	
Home	783	688	756	901	1439	881	
Overall	2288	2322	3139	4326	7655	4192	
OOP as Percentage of Household Non-food Expenditure	19.2 (7.3)	10.6 (0.9)	11.8 (0.8)	12.2 (0.7)	14 (0.9)	13.2 (1)	
OOP as Percentage of Total Household Expenditure	4 (0.4)	3.6 (0.3)	4.3 (0.3)	4.4 (0.2)	5.7 (0.3)	4.5 (0.1)	
***Catastrophic Health Expenditure (%)***							
Public	2.0	2.5	1.8	1.4	0.6	1.6	-0.224 (-0.353, -0.094)
Private	41.2	28.8	27.5	22.4	15.3	22.0	-0.190 (-0.232, -0.147)
Overall	8.4	6.7	7.9	8.1	8.1	7.9	0.0129 (-0.031, 0.057)
***Impoverishment Due to Expenditure (%)***							
Pre-payment Poverty Headcount (@ 1.25$ Int.)	13.8	12	7	4.7	1.4	6.6	
Post-payment Poverty Headcount (@1.25$ Int.)	16.2	15	10.7	7.2	4.1	9.5	
Increase in Poverty (@ 1.25$ Int.)	2.4	2.9	3.6	2.5	2.8	2.9	
Pre-payment Poverty Headcount (@2$ Int.)	42.9	38.7	31.2	21.2	9.7	25.7	
Post-payment Poverty Headcount (@2$ Int.)	45.8	41.9	35.4	25.9	14.3	29.8	
Increase in Poverty (@ 2$ Int.)	2.9	3.2	4.2	4.6	4.7	4.1	

**Table 3:** Care Utilization and Out-of-Pocket Expenditure for Delivery in 21 Districts of Haryana State, India.

Districts	Public Sector Delivery	Mean OOP Expenditure for Delivery INR (USD*)			Women Incur No Expenditure	Catastrophic Health Expenditure (%)	
	(%)	Public	Private	Home	(%)	Public	Private
Ambala	59.1	862 (15.8)	15335 (282)	944 (17.4)	63.2	0.9	7.3
Bhiwani	60.6	297 (5.5)	8223 (151)	546 (10)	85.6	1.4	12.8
Faridabad	63.2	766 (14.1)	14198 (261)	1569 (28.8)	23.4	1.3	31.4
Fatehabad	60.3	629 (11.6)	8881 (163)	422 (7.8)	41.9	0.7	12.6
Gurgaon	56.4	1428 (26.2)	17231 (317)	478 (8.8)	51.4	1.3	18.8
Hisar	58.9	569 (10.5)	8905 (164)	600 (11)	81.0	2.9	36.0
Jhajjar	72.6	1549 (28.5)	14285 (263)	483 (8.9)	65.8	2.5	20.2
Jind	60.9	261 (4.8)	8974 (165)	6 (0.1)	86.5	1.5	20.3
Kaithal	66.8	460 (8.5)	10715 (197)	614 (11.3)	63.2	1.2	39.0
Karnal	63.0	633 (11.6)	14470 (266)	407 (7.5)	63.1	0.0	22.4
Kurukshetra	64.5	65 (1.2)	11828 (217)	1161 (21.3)	94.2	0.0	9.7
Mahendergarh	77.7	560 (10.3)	11360 (209)	110 (2)	62.4	0.0	20.4
Mewat	74.2	639 (11.8)	9172 (169)	489 (9)	56.0	0.0	17.4
Palwal	70.3	671 (12.3)	10493 (193)	237 (4.4)	42.3	0.4	10.7
Panchkula	83.6	662 (12.2)	17563 (323)	17 (0.3)	76.9	0.9	7.7
Panipat	60.0	476 (8.7)	12988 (239)	3827 (70.4)	77.3	1.4	27.6
Rewari	54.8	959 (17.6)	15833 (291)	1224 (22.5)	70.2	5.2	32.5
Rohtak	67.3	1522 (28)	9868 (181)	837 (15.4)	42.7	1.8	13.3
Sirsa	66.5	295 (5.4)	8499 (156)	146 (2.7)	78.2	0.0	38.9
Sonipat	59.6	1560 (28.7)	14399 (265)	1845 (33.9)	74.6	1.6	15.8
Yamunanagar	66.8	658 (12.1)	9715 (179)	1226 (22.5)	69.4	4.3	22.6
Haryana	65.0	771 (14.2)	12479 (229)	881 (16.2)	63.7	1.6	22.0

*1 USD = 54.4 INR using the average conversion rate given by Reserve Bank of India for the year 2012–13.

### Out-of-Pocket Expenditure

Approximately 63% of the women reported no expenditure on delivery care treatment in the public sector, which is reflective of the implementation of the free cashless delivery program (JSSK). Almost 66% of women living in rural areas and 52% living in urban areas reported that they used delivery services at public sector institutions without incurring any OOP. Equivalent numbers of women incurred no expenses for delivery services (63%) irrespective of their education status. The proportion of women reporting free delivery care in the public sector was high among the poorest (68%) and low in the richest (54%) quintiles, indicating equitable targeting of the public subsidy in Haryana (Table 2). Wide inter-district variation was observed in implementation of the JSSK program, with 23% women reporting no expenditure for delivery in the public sector in Faridabad, while free delivery services were almost universal in Kurukshetra (94%) (Table 3).

We found the mean OOP expenditure on delivery in Haryana state to be INR 4,192 (USD 77), which was INR 881 (USD 16.2), INR 771 (USD 14.2) and INR 12,479 (USD 229) for home, public sector and private sector deliveries, respectively. Considerable variation was observed across the different wealth quintiles in public sector deliveries. The mean out-of-pocket expenditures for delivering in public facilities among the poorest and wealthiest quintiles were INR 633 (USD 11.6) and INR 1,024 (USD 18.8), respectively (Table 2). Significant inter-district variation in OOP expenditures was observed, particularly in public sector and home deliveries (Table 3). OOP expenditure on delivery as a percentage of households’ non-food consumption expenditure was 13.2%, and was 19.2% in the lowest wealth quintile and 14% for the wealthiest quintile (Table 2).

### Catastrophic Delivery Expenditure & Impoverishment

For women who delivered babies in public sector institutions, OOP expenditure was catastrophic for 1.6% of households (Table 2). For private sector deliveries, OOP expenditure was catastrophic for 22% households, with its prevalence as high as 41.2% among those in the poorest quintile. Overall, almost 8% households had catastrophic expenditure on institutional delivery in Haryana. OOP expenditure on delivery resulted in a 2.9% increase in the BPL population at the 1.25$ (International dollar) poverty threshold. The incidence of impoverishment was 4.1% at a threshold of 2$ (International dollar) (Table 2).

Catastrophic delivery expenditures in Haryana were inequitably higher among the poor (CI = -0.129) (Table 2). OOP financing of delivery was found to be regressive, with the poorest households having to spend much more as a proportion of their non-food expenditure (19.2%) than their richest counterparts (14%). Similarly, the poorest households faced significantly higher catastrophic expenditure on delivery at public (2%) and private (41.2%) institutions, which was significantly inequitable (CI = -0.224 (Public), CI = -0.129 (Private)). The increase in poverty as an impact of expenditure on delivery was observed to be low in poor quintiles compared with their wealthier counterparts (Table 2).

### Trends in OOP Expenditure for Institutional Delivery

According to the National Sample Survey (NSS), OOP expenditures on delivery at public health facilities in rural and urban areas of Haryana in 2004–05 were INR 2,786 and INR 1,096, respectively [35]. For women who delivered in the private sector, OOP expenditures were INR 5,240 and INR 4,520 for rural and urban areas, respectively [35]. Adjusting the NSS estimates (2004–05) for inflation using the wholesale price index, OOP expenditures on delivery were INR 2,836 and INR 7,130 (combined rural and urban) for public and private facilities, respectively, in 2004–05, which represents the pre-NRHM period. Comparing the estimates of NSS (adjusted for inflation) and our study, the OOP expenditures on delivery in the public sector decreased in the post-NRHM period by 73% in Haryana. In contrast, OOP expenditure on delivery in the private sector rose by a considerable 75% (1.75 times) in the post-NRHM period.

### Determinants of Financial Risk Protection

We found a low correlation (less than 0.3) between the independent variables used in the regression model, indicating the absence of multi-collinearity. In the model with ‘No *expenditure on delivery’* (in public sector deliveries) as the outcome variable, we found that the women living in rural areas (p < 0.001) were almost 1.6 times more likely to incur no expenses on delivery than women living in urban areas. Similarly, the women in poorer quintiles (OR = 2) and having normal deliveries (OR = 1.535) were more likely not to incur any expenses in their deliveries than their counterparts (Table 4).

**Table 4:** Factors Affecting Financial Risk Protection for Delivery Using Logistic Regression.

		No Expenditure on Delivery*			
				95% Confidence Interval for OR	
Predictors		p	Odds Ratio (OR)	Lower	Upper
Area	Rural	<0.001	1.700	1.459	1.980
	Urban	**Ref**.			
Religion	Hindu	**Ref**.			
	Muslim	<0.001	.642	.518	.796
	Christian	.870	1.030	.724	1.466
	Sikh	.365	.880	.667	1.161
Caste	SC	.134	.890	.764	1.036
	ST	.077	.495	.227	1.078
	OBC	.018	.828	.708	.968
	General/Others	**Ref**.			
Occupation	Self Employed	**Ref**.			
	Wage Employee and Others	<0.001	2.194	1.702	2.829
	Unemployed	.949	.994	.832	1.188
	Salaried Employee	.942	.994	.857	1.154
Education	Illiterate	**Ref**.			
	Literate	.462	1.047	.926	1.184
Wealth Quintile	Poorest	<0.001	1.792	1.482	2.167
	Poor	<0.001	1.722	1.441	2.057
	Moderate	<0.001	1.530	1.291	1.814
	Rich	<0.001	1.445	1.225	1.704
	Richest	**Ref**.			
BPL Status	BPL	.702	1.027	.896	1.177
	Non-BPL	**Ref**.			
Type of Delivery	Normal	.001	1.366	1.143	1.633
	C-section	**Ref**.			

*Applicable for only public sector deliveries.

Note: Ref.: Reference category

## Discussion

A number of developing countries have shown an aspiration to achieve universal health care in the 21^st^ century. India also convened a high-level expert group to develop a roadmap for UHC, which contributed to the development of the 12^th^ five-year plan [14, 15]. More recently, the Government of India has begun considering rolling out a National Health Assurance Mission (NHAM) in a phased manner in the country [36]. Despite this goal of equitable access for all, factors such as education, gender, socio-economic status, and geographical location are commonly reported to be associated with access to health care utilization in developing nations [14, 37, 38]. One of the essential components of a UHC package is providing financial risk protection to the population to avoid the need for direct payments by individuals for their health care needs. In planning for UHC, maternal and child health services are among the priority interventions for any benefit package.

We found that the OOP expenditures for delivery in public and private health facilities were INR 771 (USD 14.2) and INR 12,479 (USD 229), respectively. OOP expenditures on delivery in the public sector facilities of Haryana decreased in the post-NRHM period by 73%, while it rose by 75% (1.75 times) in the private sector during same period. This finding is indicative of the success of NRHM’s policies and programs in reducing OOP expenditures for institutional delivery in public sector facilities. Increased public health spending in the post-NRHM period and introduction of focused strategies, such as the JSY *‘conditional cash transfer scheme’* and JSSK *‘cashless delivery’*, have contributed positively toward reducing OOP expenditures and thus minimizing financial barriers. The deliveries in public sector have been subsidized through policy interventions to such an extent that the direct expenditures tend to be lower than even home deliveries where women have to incur expenditure on account of payment of fees to the birth attendant. We would like to note that the indirect expenditure on account of wage loss, or reduced productivity was not measured as part of the study.

A study that analyzed DLHS-III data found that the overall OOP expenditure for delivery (institutional and home) in Haryana was USD 65, which in our case is USD 77 (1 USD = INR 54.4) [10]. After adjusting for inflation, the estimate given by Mohanty et al. becomes USD 95 for the year 2012. Institutional deliveries during the DLHS-3 period were less than 50% and took place predominantly in private institutions (70% private share). In contrast, our study shows that over 80% of deliveries were institutional, of which almost 65% occurred in public institutions. This finding again indicates that a shift toward public sector deliveries as a result of interventions under the NRHM has resulted in lowered OOP spending and greater financial risk protection.

A study assessing expenditure on delivery during the post-JSY period in 4 districts of India’s Rajasthan state found a substantial decrease in the financial burden for delivery care due to the introduction of conditional cash transfers. JSSK enhanced this effect with cashless deliveries in public sector institutions [39]. Another study performed in North India assessing the impact of JSSK on OOP expenditures among the urban slum dweller population found a one-third reduction in OOP expenditures in the post-JSSK period [19].

Our study results show a wide variation in OOP expenditure on delivery across different socio-economic groups, regardless of place of delivery. There was a consistent increase in OOP expenditure in the public sector moving from the poorest (USD 11.6) to the wealthiest quintiles (USD 18.8), reflecting the tendency of rich women to pay more for delivery care services in a quest to obtain quality services. Other recent studies also obtain the same findings and make similar arguments [10, 40].

Prior to the launch of NRHM, utilization of public health facilities for delivery was poor. Multiple barriers, predominantly financial, obstructed the utilization of health facilities [41]. Increased public spending and focused schemes, such as JSY and JSSK, under the umbrella of NRHM have led to increased utilization of public sector health facilities. Bonu et al. 2009, using the data from the NSS 60^th^ round (2004–05), reported the utilization of public and private health facilities for delivery care services in Haryana to be 5% and 28%, respectively [11]. Similarly, Mohanty et al. 2012, analyzed DLHS-III (2007–08) data and reported that the breakdown of institutional delivery in Haryana was 15% public sector and 32% private sector [10]. Comparing these studies with our findings suggests that the NRHM interventions have resulted in a positive shift in the contribution of the public sector to overall institutional care at delivery. This shift has also been indicated in another study from Uttar Pradesh [42].

Our findings on OOP expenditure on delivery as a percentage of total and non-food annual consumption expenditures are similar to those reported by Garg and others [40]. We estimated OOP expenditures on delivery to be 4.5% and 13.2% of total and non-food consumption expenditures, respectively. Similarly, Garg et al. found OOP expenditures on delivery to be 4.8% and 10.7% of total and non-food consumption expenditures, respectively [40]. Our estimates for increase in impoverishment (2.9%) as a result of expenditure on delivery are again closer to those in the above mentioned study (3.2%). However, methodological differences lead to small differences between the two estimates. We use the international standard of poverty threshold (i.e., International 1.25$), whereas the referenced study used the poverty line given by India’s planning commission in 2001. Bonu et al. estimated the incidence of catastrophic delivery expenditure, using non-food expenditure as a reference, to be 31% for Haryana in 2004–05, whereas our study estimates the same for Haryana in 2012–13 to be 8% [11]. There are methodological differences in estimation because the other study used standard poverty cut-offs to remove the subsistence expenditure from the total consumption expenditure because NSS (2004–05) does not provide a breakdown of total household expenditure as food and non-food expenditures. Our survey collects data on both components (i.e., food and non-food expenditures); thus, it captures the variability of dietary habits and associated costs for populations across various socio-economic groups, unlike the NSSO data, and therefore adds to the robustness of the estimates. Another study from north India concluded that although absolute OOP expenditures increase with a rise in socio-economic status, even relatively low spending in the low-wealth quintile is catastrophic [27]. Second, this study also recommends the use of non-food expenditure for estimating catastrophic impact. Nevertheless, the reduced extent of catastrophic health expenditure for delivery in Haryana, compared with the NSSO estimates reported earlier, indicates a positive impact of NRHM on improving financial risk protection for maternal health care.

We found that the dominant determinants of OOP expenditure on delivery were women’s place of residence, socio-economic status, place of delivery and type of delivery. The results of multivariate logistic regression suggest that a woman is more likely to have free delivery service if she delivers in a public institution. Moreover, the odds of women not incurring any expenses are 1.5 times higher for those undergoing normal delivery than for women having Caesarean section delivery. Similar to our findings, Mohanty et al., found that women delivering in private health facilities were likely to incur costs that were 4 times higher than women delivering in the public sector and that women undergoing Caesarean section delivery were likely to incur costs that were 6 times higher than women having normal delivery [10].

The major strength of our study is its large dataset for a very recent period. We interviewed 12,191 women with their deliveries occurring between September 2011 and March 2014. We could not find any study with such a large and representative sample that covered a recent period. Earlier studies have utilized NSSO (2004–05), NFHS (2005–06), or DLHS (2007–08) data, which are dated considering the policy initiatives undertaken under the NRHM from 2005 onward. However, we note certain design limitations in attributing the causality of positive findings regarding institutional delivery care to NRHM interventions. Our study design lacks a control group, which limits the strength of causal attribution.

However, comparing our findings on the extent of public sector institutional delivery, free care in the public sector, OOP expenditure and catastrophic expenditure to those reported for the pre-NRHM period offers some indication of the beneficial effects of program interventions. Moreover, all the findings are in a similar direction (i.e., increase in utilization, reduction in absolute OOP spending and increase in financial risk protection).

While it is encouraging to observe an increase in institutional care at delivery with reasonably high financial risk protection, it is important to assess the quality of such institutional deliveries in public sector facilities. This will determine the extent to which it is likely to bring about a reduction in maternal and early neonatal mortality. Some early evidence suggests that initiation of conditional cash transfer scheme such as Janani Suraksha Yojana (JSY) led to a reduction of 3·7 perinatal deaths per 1000 pregnancies and 2·3 neonatal deaths per 1000 live births [12]. A number of other changes in the programs for increasing institutional deliveries have happened since the earlier analysis. Hence it is important to assess, not only changes in the institutional deliveries brought about by this increased public investment, but also impact on the health outcomes.

### Conclusion and Recommendations

Based on the findings of the present study, we conclude that there is considerably high coverage of institutional delivery in Haryana and at a cost that does not impose significant financial barriers. Almost two-thirds of the total institutional deliveries take place in public sector facilities, of which, in turn, two-thirds are free of any out-of-pocket expense. Moreover, the utilization of public sector facilities is equitable in Haryana. Together, these are significant findings that indicate that the public sector is capable of providing universal health care provided that sufficient funds are available and are backed by political and administrative commitment. Variations in the performances of various districts must be addressed. More research is recommended to assess the causal impacts of the interventions introduced in the post-NRHM period in universalizing institutional delivery, and any change in maternal and early neonatal mortality which has been brought about by this increased public investment for institutional delivery.
